# The influence of cancer on a forensic age estimation tool

**DOI:** 10.18632/aging.206281

**Published:** 2025-07-17

**Authors:** Charlotte Sutter, Daniel Helbling, Cordula Haas, Jacqueline Neubauer

**Affiliations:** 1 Zurich Institute of Forensic Medicine, University of Zurich, Zurich 8006, Switzerland; 2 Onkozentrum Zurich, Zurich 8038, Switzerland

**Keywords:** forensic age estimation, age prediction, cancer, DNA methylation, age acceleration

## Abstract

The use of epigenetic clocks for measuring age acceleration in the field of cancer research has been a common practice for many years. In forensic genetics, DNA methylation can be used to estimate the age of a stain donor. As lifestyle and disease can alter a person’s methylation profile, the accuracy of forensic age estimation tools might decrease compared to the chronological age when estimating a person affected by cancer. In our study, we applied the VISAGE enhanced age estimation tool on blood samples from cancer patients suffering from a variety of cancer entities, including solid and hematologic tumours. A comparison of the age estimation errors between the cancer patients (n = 100) and a healthy control cohort (n = 102) revealed small statistically significant differences and a tendency towards age acceleration in the blood of these patients. Although this study showed that in patients with aggressive cancers (like CLL or AML) estimation accuracy is clearly decreased, for most entities the observed differences were subtle and an analysis of individual CpG sites did not reveal strikingly different methylation patterns. Conclusively, age estimation on blood stains from cancer patients might not result in significantly higher estimation errors, except for very aggressive forms of cancer.

## INTRODUCTION

Over the past few decades, epigenetic research has opened up vast potential for applications across many biomedical fields. One of these is the ability to infer a person’s age from their DNA methylation (DNAm) profile at a given time in life [1, 2]. Following the original epigenetic clock developed by Horvath [1], many fields have adapted such age clocks for their particular needs [3–9].

In forensic genetics, the possibility to predict the age of a stain donor from biological material collected at a crime scene has received much attention in recent years [10, 11]. When traditional approaches like STR profiling do not produce a hit in a DNA database, phenotype information can help narrow down the pool of suspects. Phenotyping includes the prediction of externally visible characteristics, such as pigmentation traits (eye, hair and skin colour), biogeographic ancestry, as well as age estimation which can help to determine someone’s appearance. [11, 12]. The analysis of DNAm markers is currently the method of choice for estimating donor age [13].

Due to low DNA quality and quantity, which are commonly limiting factors in forensic samples [11], forensic age estimation tools typically examine only few markers and target CpG sites [7, 9, 11, 14–18] compared to traditional first- and second- generation epigenetic clocks (e.g. Horvath’s clock, Hannum’s clock, PhenoAge, GrimAge, etc. [1, 19–24]). Ideally, markers for forensic tools should be robust to environmental factors, and their methylation patterns highly correlated with age. In addition, forensic age estimation tools mostly comprise target sites and statistical models that are specifically selected and designed for a particular forensically relevant body fluid, such as blood or saliva [9]. This is due to the cell type and tissue specificity of DNAm patterns, which requires the investigation of cell type specific target sites to achieve the highest possible age estimation accuracy [12].

DNAm is commonly known to be heavily influenced by environmental factors [25, 26]. Other studies have shown that e.g. smoking, physical activity or diet as well as intrinsic factors like diseases can strongly influence the observed DNAm patterns [2, 20, 27–29]. This poses a problem for forensic applications as it is crucial that the generated age estimation matches the person’s chronological age as closely as possible [11]. Therefore, influencing factors on DNAm patterns need to be addressed as a part of the validation of forensic age estimation tools for use in forensic casework. Several studies have already investigated different potentially influencing factors, such as alcohol consumption or smoking, with regard to forensic age estimation tools [27, 28, 30]. However, many factors still need to be thoroughly elucidated in a forensic context. One of these is the influence of cancer. This has been briefly addressed in two studies, one showing that the estimated age of patients with chronic lymphatic leukaemia (CLL) is significantly higher than in healthy controls [30], while another study found no differences between patients with colorectal cancer aged between 50 and 60 years [31]. However, a comprehensive investigation of different tumours and their potential impact on forensic age estimation tools is still missing.

Cancer is the second leading cause of death worldwide [32] and is therefore very common in the general population. Age is one of the strongest risk factors for developing a cancerous disease [33–38]. Thereby, molecular mechanisms reflecting the biological aging process have been strongly implicated in the development and risk of cancer [33]. Among these mechanisms, DNAm has long been known to show altered patterns in most types of cancer compared to healthy tissue [39, 40]. Normally, the majority of CpG sites in the human genome are methylated [41] with the exception of CpG islands in promotor regions [42–44]. These patterns are maintained by three methyltransferases (DNMT1 for DNAm pattern maintenance and DNMT3a and 3b for *de novo* methylation [41]), and ten-eleven translocation (TET) proteins, responsible for DNA demethylation [44]. However, in cancerous tissue promotor CpG sites of tumour suppressor genes tend to be hypermethylated, especially when targeted by polycomb protein complexes [33, 35, 42, 45], thereby silencing these genes. In addition, repetitive elements that are normally methylated appear to undergo increasing demethylation in cancerous tissue [35, 42, 46]. One of the reasons for these aberrant methylation patterns, that are actually related to the normal aging process, is a decrease in activity of DNMT1 over the course of a person’s life. This causes passive demethylation and thereby promotes carcinogenesis [42]. In addition, it has been proposed that the cumulative number of cell divisions over time (another natural event of aging) and the resulting alterations in DNAm patterns contribute considerably to tumourigenesis [35].

Due to the increasing body of knowledge about the role of DNAm in carcinogenesis as well as its correlation with disease progression, numerous studies have investigated the possibility of using epigenetic clocks to predict disease outcome, treatment success or the development of comorbidities [47–52]. For this purpose, first- and second- generation epigenetic clocks [1, 19–24] targeting 100s to 1000s of CpG sites, as well as newly developed clocks specifically designed for cancer research [49], have been applied to tissue samples (mostly blood) from cancer patients. Most of these studies have in common that they show an increased biological age compared to the chronological age of cancer patients and even reliably predict potential secondary conditions (e.g. [52]). In addition to the disease itself, therapeutic interventions like chemotherapy have been shown to cause lasting alterations in DNAm patterns [53]. Even after successful treatment and in complete remission, cancer survivors might show significant age acceleration.

Although these findings are important advances in the field of cancer research, paving the way for new potential treatment targets and possibilities for disease prediction and intervention [36, 42, 48], alterations in the human methylome due to cancer might have negative implications for the field of forensic genetics. Not only is it crucial that the estimated age corresponds as closely to the chronological age as possible, independent of any medical condition, but also that the inference of health-related information is avoided. As forensic age estimation tools use a much smaller number of target CpG sites than clinical epigenetic clocks [7, 9, 14], cancerous diseases might not at all or only occasionally affect exactly these sites. Still, there is a possibility to observe higher estimation errors in comparison to the respective chronological age caused by the influence of cancer and its treatment.

In this study, we apply an existing forensic age estimation tool for blood [7] to whole blood samples from patients with various tumour types. Our aim is to show whether the presence of cancer in different stages and the received treatment affect the tested target CpG sites and thus the accuracy of this age estimation tool. As the latter includes some of the most common markers (*ELOVL2* [54], *KLF14* [55], etc.) and target CpG sites used in many forensic age estimation tools, our findings will be applicable to the field of methylation-based forensic age estimation in general. Cancer is an important condition to investigate in this context due to its high prevalence in the general population and therefore there is an increased likeliness to encounter stain donors affected by some form of cancer in forensic casework. Our study is among the first to show whether it might be necessary to account for the influence of cancer on forensic age estimation tools in order to enhance estimation accuracy as much as possible.

## RESULTS

### Study cohort

For the cancer cohort, blood samples were collected from 100 patients (age range [y] = 25.46 - 93.15, mean = 65.88) with a variety of malignancies. For each patient, the following meta data were enquired: chronological age, sex, cancer entity, current cancer stage, current treatment and previous treatment (more than 3 months ago). Table 1 and Supplementary Table 1 show detailed summaries of the cancer cohort meta data. The control cohort of this study comprised blood samples from 102 individuals collected at the Blood Donation Centre Zurich (age range [y] = 20.87 - 73.16, mean = 46.12) with an even age distribution between the ages of 20 and 70 years. Each decade included between 18 and 23 individuals in addition to two individuals over 70 years of age. The age and sex distribution of the control cohort can be found in Supplementary Table 1.

**Table 1:** Meta data of the entire study cohort, CT = current treatment, PT = previous treatment.

**Subgroups**	**Subgroups detailed**	**Cancer stage**	**CT**	**PT**	**MAE**	**n**
Controls (n = 102)	Healthy	Healthy	Healthy	Healthy	2.72	102
Hematologic tumours (n = 25)	Lymphoma	Not applicable	Yes	Yes	16.00	3
				No	2.86	3
			No	Yes	4.22	3
				No	8.14	3
	Myeloma	Not applicable	Yes	Yes	7.96	3
	Others	Not applicable	Yes	Yes	14.36	1
				No	14.74	3
			No	Yes	20.08	1
				No	16.44	5
Solid tumours (n = 75)	Breast	Stage 1-3	Yes	Yes	5.18	7
		Stage 4	Yes	Yes	5.43	2
	Gastrointestinal	Stage 1-3	Yes	Yes	3.78	16
				No	7.41	10
		Stage 4	Yes	Yes	4.70	4
				No	3.29	4
	Genitourinary	Stage 1-3	Yes	Yes	3.01	6
				No	4.29	2
			No	Yes	0.46	1
				No	0.71	1
		Stage 4	Yes	Yes	11.54	1
				No	2.30	3
			No	Yes	0.02	1
	Lung	Stage 1-3	Yes	Yes	3.84	4
				No	7.10	1
		Stage 4	No	Yes	7.59	1
				No	2.55	1
	Prostate	Stage 1-3	Yes	Yes	7.63	9
				No	11.92	1

Contrary to the control cohort, the cancer cohort did not consist of an even distribution of samples in each age category. A statistical comparison of the chronological age distribution between the three subgroups showed that for both cancer groups, the age distribution was statistically significantly different from that of the control cohort (Supplementary Table 2).

### Age estimation

All blood samples from the entire study cohort were estimated with the age estimation tool published by Woźniak et al. [7, 11] which requires the methylation percentages of six target CpG sites in *ELOVL2*, *TRIM59*, *PDE4C*, *KLF14*, *MIR29B2C* and *FHL2*. The mean absolute error (MAE), the mean error (ME) and root mean square error (RMSE) were used to assess the age estimation accuracy within specific subgroups of the study cohort.

When the cancer samples were divided into two subgroups (hematologic tumours (n = 25) and solid tumours (n = 75)), the MAE and RMSE of the solid tumours were 4.98 and 6.41 years, whereas the MAE and RMSE of the hematologic tumours were 11.14 and 16.32 years, respectively. In addition, the MAE was calculated for all possible subgroups taking into account the available meta data (Table 1). The MAE and RMSE for all control samples were 2.72 and 3.36 years, respectively. A scatterplot depicting the chronological vs. the estimated age of all samples (n = 202) was generated as well (Supplementary Figure 1).

The absolute errors of the solid tumour group and the hematologic tumour groups were statistically significantly different from the control cohort (p-value solid vs. control = 0.0003; p-value hematologic vs. control = 0.00001; Wilcoxon test).

As the control cohort only included individuals up to the age of 73 years, and the used age estimation tool was developed and tested in individuals below 75 years of age [7], all cancer patients in this study over the age of 75 years (n = 31) were excluded from most of the following analyses. All individuals above 75 years of age are discussed individually further below.

**Figure 1:**
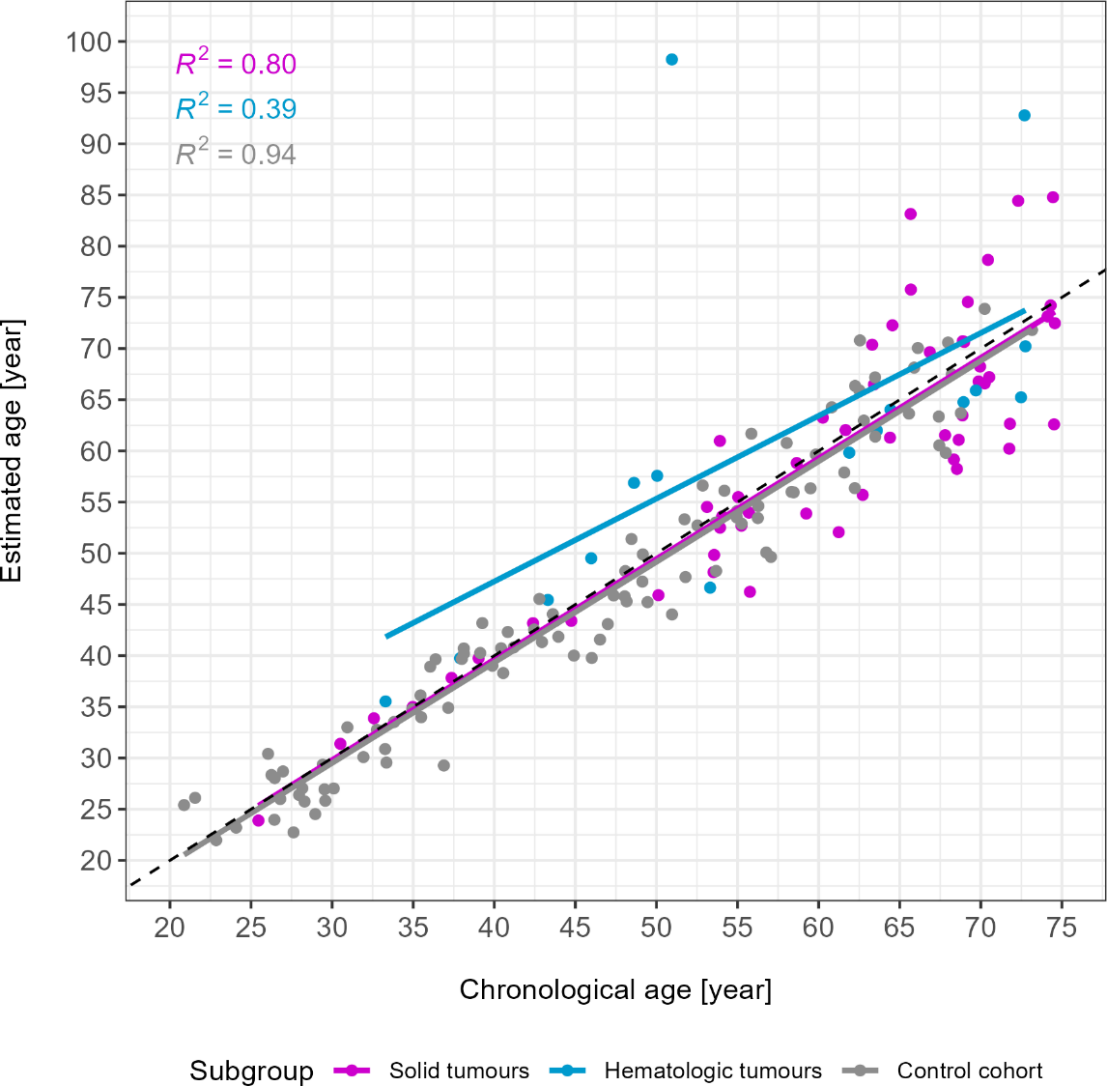
**Age estimates of the u75 study cohort and control samples.** Solid tumours are depicted in magenta (n = 53), hematologic tumours in blue (n = 16) and the control cohort in grey (n = 102). R^2^ of each subgroup are depicted in the upper left corner. Two outliers were identified visually. The grey dotted line is the line of identity. MAE in years: 4.66 (solid tumours), 7.59 (hematologic tumours), 2.72 (control cohort).

Repeating the analysis of MAE and RMSE of the under 75 (u75) cancer subgroups (n = 69) showed an MAE and RMSE of 4.66 and 6.16 years, respectively, for the solid cancers (n = 53), and an MAE and RMSE of 7.59 and 13.54, respectively, for the hematologic cancers (n = 16) (Figure 1).

As a result of excluding individuals above 75 years of age, the comparison of the absolute errors between the cancer subgroups and the control cohort yielded weak statistically significant differences (p-value solid vs. control = 0.02; p-value hematologic vs. control = 0.03; Wilcoxon test).

Because of the lack of age matching between the cancer and control groups (u75 cohort), we additionally used logistic regression to evaluate whether absolute age estimation errors were truly associated with cancer status even when controlling for chronological age and sex. Disease status (healthy or affected by cancer) was used as the dependent variable and absolute estimation error, chronological age and sex were used as independent variables (Supplementary Table 3). This analysis showed that higher age estimation errors were not significantly associated with cancer status even after adjusting for age and sex, confirming our previous results. Chronological age was significantly associated with a lower probability of being in the cancer group, indicating again the previously reported age-related imbalance in the dataset. Additionally, sex was observed as a significant predictor which is likely also explained by the imbalance of males and females present in the study cohort (Supplementary Table 1).

A more detailed analysis of the age estimation errors in the different age groups provided additional insight into where statistically significant differences between either of the two case groups (solid tumours and hematologic tumours) and the control cohort could be found (Table 2). In short, when comparing the control group and the solid tumour group, statistical differences in age estimation errors (p-value = 0.0352, Wilcoxon test) were found only in the 30 - 39 years age category. When comparing the hematologic tumour group with the control group, only the age category 50 - 59 years showed statistically significant differences in age estimation errors (p-value = 0.007, Wilcoxon test). A summary of the percentage of samples in the solid or hematologic tumour subgroups with age estimation errors above the MAE or RMSE value of the control cohort in the respective age category can be found in Supplementary Table 4.

**Table 2:** MAEs/RMSEs per age category in each sample subgroup (SvsC = solid tumours vs control group; HvsC = hematologic tumours vs. control group).

**Age categories [y]**	**Control cohort**	**Solid tumours**	**Hematologic tumours**	**p-value SvsC**	**p-value HvsC**
20.0 – 29.9	2.49 / 2.91 (n = 18)	1.56 / 1.56 (n = 1)	-	0.632	-
30.0 – 39.9	2.21 / 2.77 (n = 20)	0.68 / 0.80 (n = 5)	2.05 / 2.06 (n = 2)	0.035*	1.000
40.0 – 49.9	2.13 / 2.73 (n = 23)	1.07 / 1.10 (n = 2)	4.65 / 5.34 (n = 3)	0.540	0.134
50.0 – 59.9	3.07 / 3.76 (n = 21)	3.34 / 4.33 (n = 13)	20.50 / 27.90 (n = 3)	0.917	0.007*
60.0 – 69.9	3.90 / 4.47 (n = 18)	5.87 / 7.07 (n = 21)	2.40 / 2.78 (n = 5)	0.213	0.257
70.0 – 74.9	2.49 / 2.47 (n = 2)	6.67 / 8.03 (n = 11)	9.95 / 12.40 (n = 3)	0.410	0.400
> 75	-	5.73 / 6.99 (n = 22)	17.40 / 20.40 (n = 9)	-	-

* p-value < 0.05.

Two outliers (more than 15 years age estimation error) in the hematologic tumour subgroup were visually identified (chronological age = 50.95 years and 72.70 years, Figure 1). When excluding these samples from the hematologic tumour group, an overall MAE of 3.86 was obtained. A comparison between the control cohort and the hematologic tumour group without these two outliers revealed a p-value of 0.13 (Wilcoxon test). In the analysis of the individual age decades, excluding the outliers resulted in a change of p-values in the groups 50 – 59 years (p-value = 0.047) and 70 – 75 years (p-value = 0.67).

Mean errors were calculated in addition to the MAE per age decade in order to evaluate whether chronological age was predominantly over- or underestimated in the two cancer subgroups (Supplementary Table 5). While most of the solid tumour patients’ age was slightly underestimated, age of the hematologic patients was mostly overestimated.

### Methylation pattern analysis of target CpG sites

The methylation patterns of the six target CpG sites of the used age estimation tool were investigated to identify biological differences between the control and cancer groups at a CpG site-specific level.

**Figure 2:**
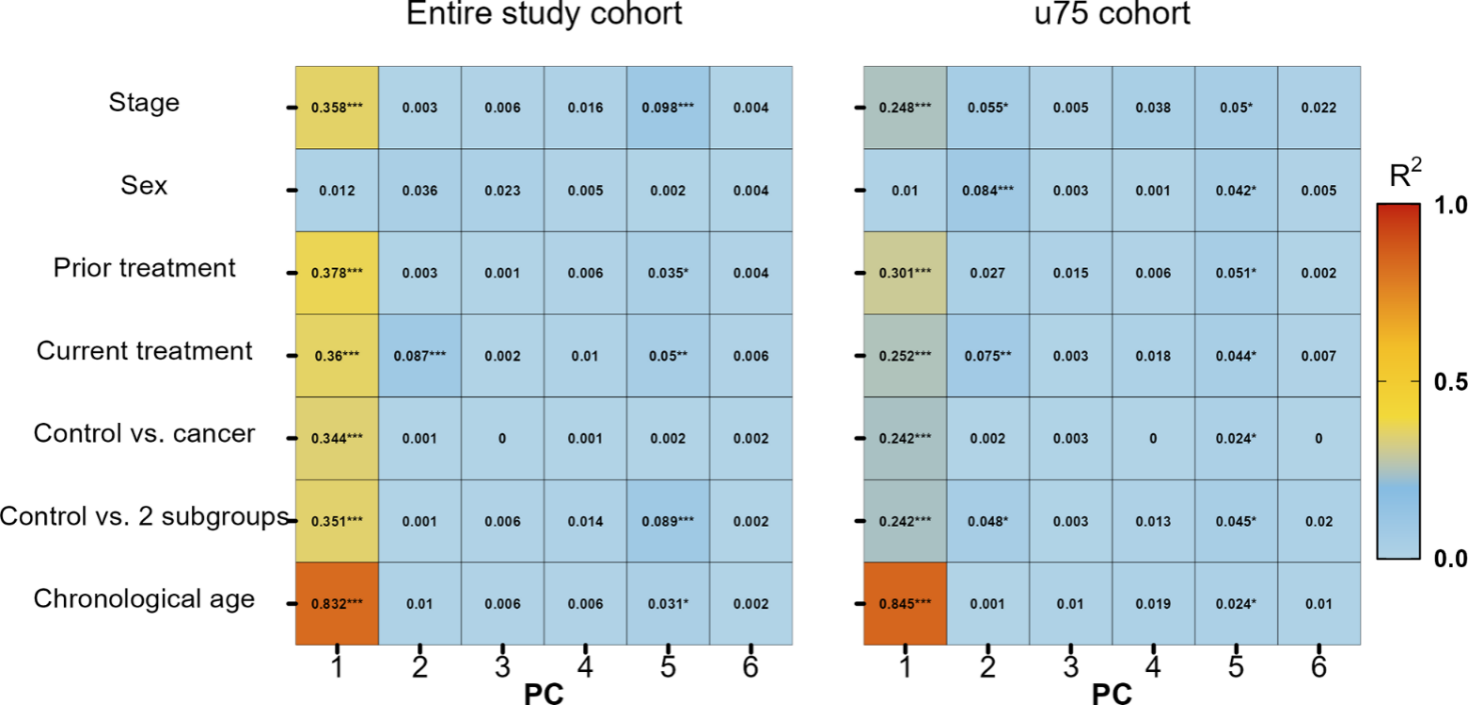
**Variable association of stage, sex, previous treatment, current treatment, control vs. all cancer patients, control vs. two subgroups (solid and hematologic tumours) and chronological age with principal components (PC) 1 to 6.** * p-value ≤ 0.05, ** p-value ≤ 0.01, *** p-value ≤ 0.001.

First, principal components (PCs) generated on beta values normalized for chronological age and their association with available meta data variables were assessed for both the entire cohort and the reduced u75 cohort (Figure 2). In the entire cohort, it is clearly visible that PC1 was the most affected of the first six PCs and that chronological age had the strongest influence (p-value = 2.29 × 10^-79^, R^2^ = 0.83). Still, all other variables except sex also had at least a moderate effect on PC1. When looking at the u75 cohort, the strong influence of chronological age on PC1 persisted (p-value = 2.64 × 10^-70^, R^2^ = 0.84), while all other variables showed weaker associations with this and all other PCs.

In addition, principal component analysis (PCA) plots were generated to investigate whether normalized beta values clustered according to any available meta data. No clear clustering was found between controls and cancer cases, treatment or cancer stage (Supplementary Figure 2).

**Figure 3:**
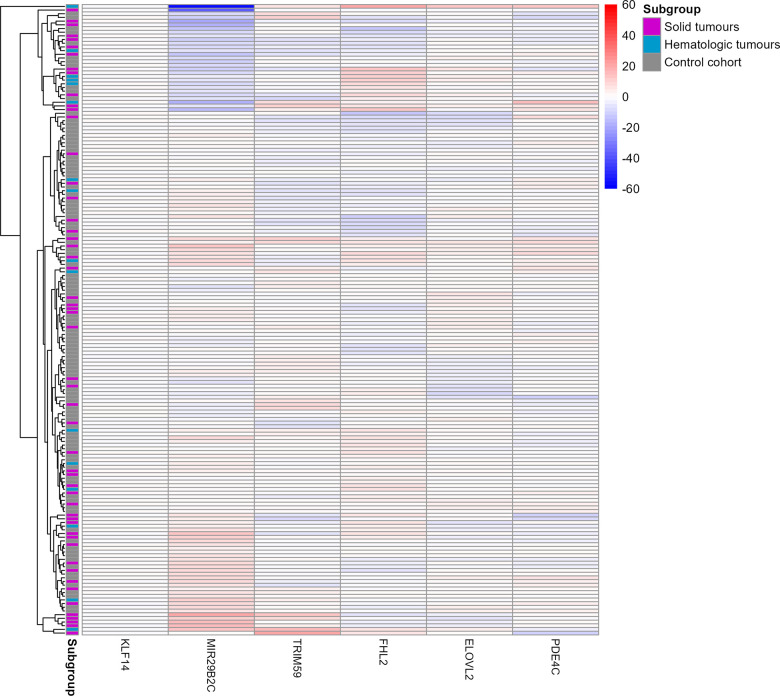
**Heat map of the six target CpG sites used in the age estimation tool in the u75 cohort and controls.** Methylation beta values are adjusted for chronological age. The labels on the x-axis refer to the respective target CpG site in each marker. The two cancer patients Cancer033 and Cancer042 in the two top rows showed a differential methylation in the *MIR29B2C* target CpG site. Red colouring indicates higher beta values and blue colouring indicates lower beta values. Rows indicating samples from the three subgroups are indicated in grey (control cohort), blue (solid tumours) or magenta (hematologic tumours).

Further evidence for only weak differences between methylation beta values of the control and cancer subgroups, independent of cancer, stage or treatment was obtained by a heat map (Figure 3). Methylation beta values were statistically adjusted for the respective chronological age via linear regression. Only two cancer patients (one solid cancer and one hematologic cancer) were identified to have considerably different beta values in the *MIR29B2C* target site. One of them even clustered apart from all other samples.

### Outliers

As can be seen from both the age estimation scatterplot (Figure 1) and the beta value heat map (Figure 3), three cases in the u75 cohort behaved differently from all other samples and had age estimation errors greater than 15 years. Firstly, Cancer033, a solid gastrointestinal tumour, and Cancer042, a CLL, displayed observable differences in the beta value of the *MIR29B2C* target site. This was also reflected in the respective age estimates, with Cancer042 in particular being overestimated by almost 50 years (chronological age [y]: 51.0, estimated age [y]: 98.2) and Cancer033 being overestimated by almost 20 years (chronological age [y]: 65.7, estimated age [y]: 83.1). Cancer111, another CLL, did not show noticeable differences in beta values in the heat map, but its age estimate exceeded the chronological age by 20 years (chronological age [y]: 72.7, estimated age [y]: 92.8).

### Cancer patients above 75 years of age

All cancer patients older than 75 years of age (n = 31), who had been excluded from the previous analyses, were reinvestigated separately for significantly different methylation beta values in the six investigated target CpG sites.

**Figure 4:**
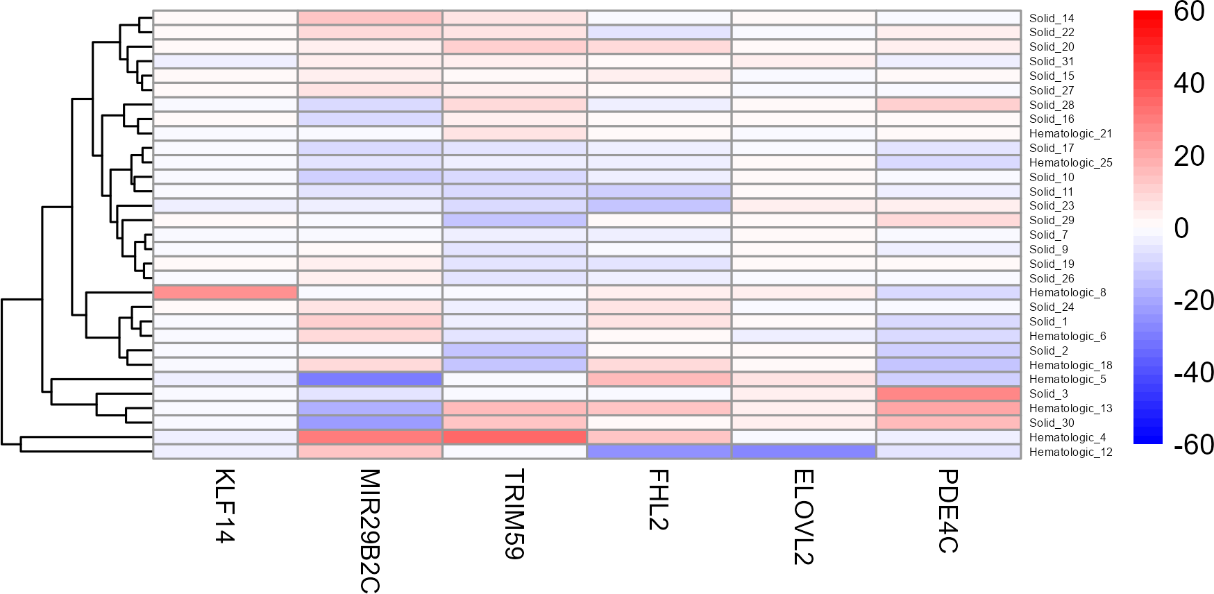
**Heat map of the six target CpG sites used in the age estimation tool in all cancer patients older than 75 years.** Methylation beta values are adjusted for chronological age. The labels of the x-axis refer to the respective target CpG site in each marker. Cancer022 (called Hematologic_4) and Cancer041 (called Hematologic_12) are the two bottom rows, clustering apart from all other samples. Red colouring indicates higher beta values and blue colouring indicates lower beta values.

For this purpose, another heat map was generated using the age-corrected beta values of these 31 patients (Figure 4). Although no clear clustering was observed, it is noteworthy that the hematologic tumours seem to be slightly more similar in their beta values than the solid tumours. Interestingly, two hematologic cases (Cancer022 and Cancer041), both suffering from an acute myeloid leukaemia (AML), clustered apart from all other samples.

### Methylation pattern analysis of all sequenced CpG sites

To get a comprehensive picture of the methylation patterns at all CpG sites (n = 44) within the amplicons sequenced with this age estimation tool, the variable association and heat map was repeated on this extended data in the u75 cohort. The variable association with the different PCs (Supplementary Figure 3) again revealed that chronological age is most associated with the first six PCs.

The heat map revealed one case that clustered apart from all other cancer cases and controls, namely Cancer042, which already clustered separately in the previous analysis of only the six target CpG sites (Supplementary Figure 4).

**Figure 5:**
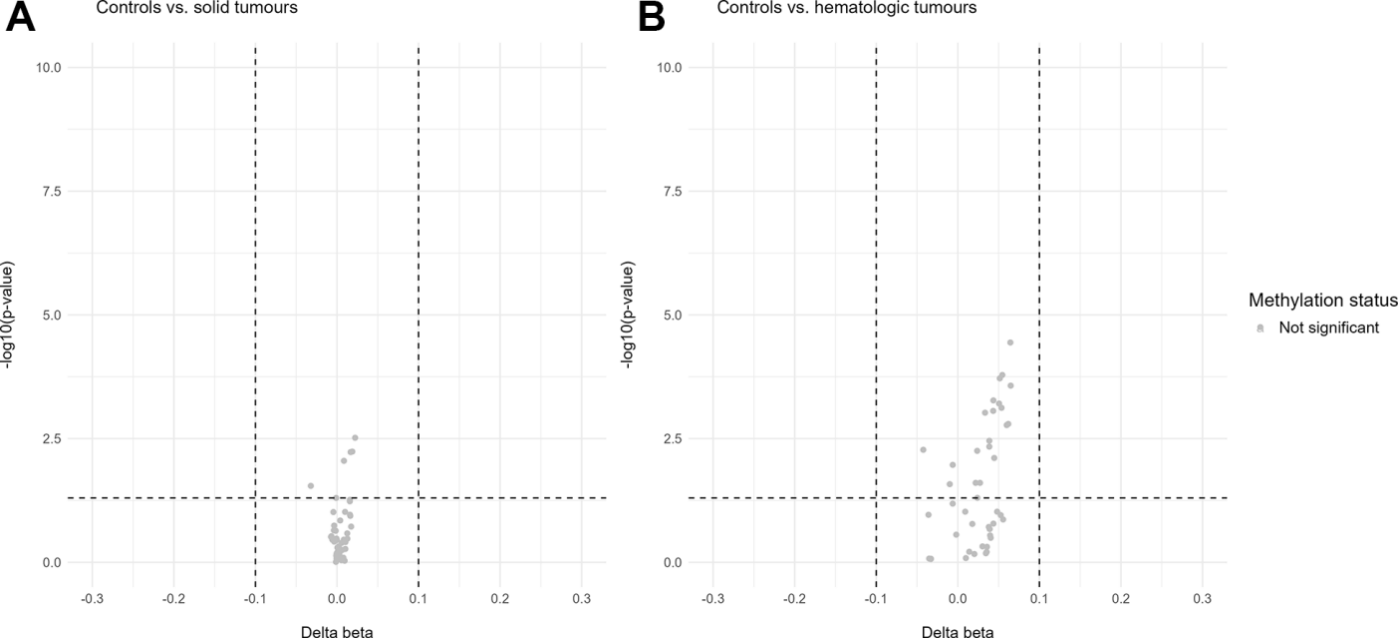
**Volcano plots of differentially methylated sites in the u75 cohort.** Hypomethylation is defined as delta beta values below -0.1 and p-values below 0.05 and hypermethylation as delta beta values above 0.1 and p-values below 0.05. (**A**) shows no differentially methylated sites between the control cohort and the solid tumours. (**B**) shows no differentially methylated sites between the control cohort and the hematologic tumours.

In addition, volcano plots were generated for the u75 cohort on the data of all 44 sequenced CpG sites, normalized for chronological age (Figure 5). The cancer cohort was again divided into solid and hematologic tumours for a more discriminative analysis of differentially methylated sites in the two different subgroups of malignancies. For neither of the two comparisons (solid vs. controls and hematologic vs. controls) differentially methylated sites were found.

## DISCUSSION

In this study, we aimed to show whether a typical DNAm-based forensic age estimation tool (few target CpG sites in strong age-associated markers) is affected by various cancer entities, their stage or treatment. Although numerous clinical studies have already investigated the effect of different types of malignant tumours on epigenetic clock measurements and how this can be exploited for clinical purposes, this study is, to the best of our knowledge, the first to comprehensively investigate whether forensic age estimation tools are affected by a range of tumour types.

As the aim of age estimation of a stain donor in a forensic context is always to estimate the chronological age of the person in question as accurately as possible [11], it is crucial to know which factors influence age estimation accuracy and to what extent [27, 28]. In the field of forensic genetics, it is often not possible or even forbidden to obtain knowledge about potential health problems of a given stain donor. For forensic age estimation tools, the only important characteristic is a high accuracy of the obtained age estimates. Due to the extensive amount of research done in the field of cancer and epigenetics, there is already a lot of evidence that cancerous diseases cause larger discrepancies between chronological and estimated age in epigenetic clocks like Horvath’s original clock [1, 2] or other second-generation clocks (GrimAge, PhenoAge, etc. [19–21]). Consequently, it can be assumed that the DNAm patterns investigated with forensic age estimation tools should also be influenced by cancerous diseases. This has indeed already been shown in a study investigating the DNAm signature of patients with hematologic malignancies [30].

For this study, we compared the age estimates of 100 patients with different cancer entities with a control group of 102 healthy individuals. It is noteworthy that the healthy control cohort was collected at a blood donation center, indicating that this cohort might have been slightly fitter and healthier than an average snapshot of the general population (no diseases, no sickness, good general condition). Within the cancer cohort, we excluded 31 patients with a chronological age above 75 years, not only because of the statistical model developed for u75 individuals [7], but also because this age group (> 75 years) is not considered the primary target age group in a forensic context. A distinction within the u75 cancer patients between solid tumours (comprising breast, gastrointestinal, genitourinary, lung and prostate tumours) and hematologic tumours (including lymphomas, myelomas and all other hematologic malignancies) revealed a slight statistically significant difference between the estimation errors of these two groups (solid tumours MAE [y]: 4.66, p-value = 0.02; hematologic tumours MAE [y]: 7.59, p-value = 0.03) and the control cohort (MAE [y]: 2.72). To better understand where the small statistically significant differences occur, an analysis of the age estimation errors in different age categories was performed. This showed that in each of the two comparisons (solid tumours vs. controls, hematologic tumours vs. controls) statistically significant differences were only observed in one particular age decade. Specifically, in the age decade of 30 - 39 years, age estimation errors were significantly lower in the solid tumour group than in the control group. In the age decade of 50 - 59 years, age estimation errors were significantly higher in the hematologic tumour group than in the control group. These statistically significant differences in the 50 - 59 age decade persisted even when the two previously identified outliers were excluded. However, it is noteworthy that the number of samples differed considerably between the control group and the solid or hematologic tumour groups in both age decades, where statistically significant differences in the age estimation error were observed. It is therefore possible that these differences are a stochastic observation. Larger sample sizes would be needed to get a fully accurate and reliable picture of how different the age estimates in these two age decades really are.

Interestingly, an analysis of the ME instead of the MAE showed that the age of solid tumour patients was predominantly underestimated, while the age of hematologic tumour patients was mostly overestimated. For solid tumour patients, these findings are counterintuitive and more samples, especially in younger patients, would be needed to evaluate whether this trend persists. For hematologic tumour patients, a slight age acceleration seems logical and is in line with previous research [30].

Within the solid tumour group, the variance in age estimates was relatively small with an R^2^ of 0.80, while the hematologic group had a much smaller R^2^ (0.39). This was likely caused by the two outliers identified in the hematologic subgroup. Their ages were overestimated by approximately 20 and 50 years, respectively. This is considerably higher than the other estimation errors, which ranged from 0.40 to 8.27 years in the u75 hematologic subgroup. Both patients suffer from CLL. In this form of leukaemia, B lymphocytes accumulate in the peripheral blood, bone marrow and secondary lymphoid tissues [56, 57]. It is the most prevalent form of leukaemia in adults in the western world [56]. As methylation patterns have previously been shown to contribute to CLL disease outcome [57], this could explain the observed high age estimation errors, which may be caused by underlying differential methylation patterns in both patients. Interestingly, an exclusion of these two outliers resulted in a non-statistically significant p-value (0.13) for the comparison of age estimation errors between the hematologic tumour group and controls. Notably, these two individuals were the only CLL patients in our u75 cancer cohort. However, given the prevalence of this entity it was considered important to include these samples in the overall analysis of the u75 hematologic tumour subgroup. The inclusion of more CLL cases might have strengthened the observed tendency towards age overestimation especially in this entity, as previously reported in [30], and should be considered in future studies.

Given the large body of clinical literature identifying age overestimation (often referred to as age acceleration) in cancer patients [20, 21, 47, 50, 51, 58], it is not surprising that our study also found at least weak evidence of age acceleration in cancer patients. To get a better understanding of the effects of cancer on the here used age estimation tool, several analyses were performed on the raw methylation beta values in order to see whether changes at a CpG site level could be identified. Interestingly, such changes in response to either environmental factors or disease-related interventions have been occasionally observed before in some of the here investigated markers. For instance, *MIR29B2C* was shown to be hypermethylated in recipients of allogeneic hematopoietic stem cell transplantation [59], while methylation in *PDE4C* was reduced through a low-fat diet [60]. *TRIM59* and *KLF14*, on the other hand, appear to be hypermethylated in elite athletes [61]. In our study, methylation of the six target CpG sites did not seem to differ in the u75 cohort between the two cancer subgroups or the control cohort, regardless of cancer entity, stage or treatment. There were again two exceptions, one cancer case that had already been previously identified due to its 50-year absolute estimation error and another case, a gastrointestinal tumour, whose methylation pattern differed slightly at one of the six target CpG sites compared to all other samples. Generally, it is most likely that too few target CpG sites were examined with this age estimation tool to capture highly altered methylation patterns and a broader analysis of a large number of CpG sites would be required to observe significant alterations.

The age estimation tool used for this study does not allow an exhaustive analysis of the human methylome due to the limited number of sequenced genomic regions (eight amplified regions in the genes *ELOVL2*, *TRIM59*, *FHL2*, *PDE4C*, *MIR29B2C*, *EDARADD*, *KLF14* and *ASPA* [7]). Still, all available sequenced CpG sites (n = 44, including the six target CpG sites of the age estimation model) were also examined to see whether an extended analysis might provide further insights into the effect of cancer, its stage or its treatment. Considering that all eight genes or their associated proteins [62–69] have been reported to be involved in tumourigenesis, it was assumed that at least some of these markers might show differential methylation at several of their CpG sites. However, similar to the investigation of the six target CpG sites, all 44 available sites showed only minor differences in methylation patterns in a heat map between controls and cancer patients. Interestingly, these results were supported by a volcano plot analysis of beta values normalized for chronological age. No hyper- or hypomethylated CpG sites were identified in the solid tumour or the hematologic tumour subgroup when comparing them to the control cohort. It is possible that the standard definition of differential methylation used here (delta beta values above 0.1/below -0.1 and p-values below 0.05) was too stringent for this cohort. In addition, the individual CpG sites might have only been slightly affected by cancer, resulting in significant differences in the estimation accuracy between the groups while still not observing significantly differentially methylated CpG sites.

In the analysis of all 44 available CpG sites, only one cancer case stood out in the heat map analysis. This case was already identified as being an outlier due to its absolute age estimation error of 50 years. As described above, this patient suffers from CLL. Considering that the influence of CLL on methylation has been suggested to be rather heterogeneous between patients [57], this might be the reason why this observed difference in methylation pattern was only present in one CLL case in our study cohort and not in both.

The initial cohort of this study also included samples from 31 cancer patients aged between 75 and 95 years. These samples were excluded from most of the analyses for several reasons. Firstly, the control cohort did not include individuals above the age of 75, therefore, a direct comparison between the control and cancer cohorts would not have been possible. Secondly, the training cohort of the used statistical age estimation model did not include individuals older than 75 years [7], therefore the model is not calibrated for accurately estimating age in older individuals. Lastly, methylation patterns are known to become increasingly variable in the elderly due to the accumulation of environmental and genetic factors [11, 13]. Therefore, in a cohort of individuals above 75 years of age, it becomes very difficult to distinguish between an increased variability in estimation accuracy due to old age or other factors such as cancer burden. Still, a preliminary analysis of these 31 cancer patients revealed two patients in particular whose methylation patterns were considerably different at some of the six target CpG sites compared to the remaining 29 cases. Both patients suffer from AML, which is caused by uncontrolled proliferation of clonal hematopoietic cells. With a very rapid progression and a 5-year overall survival prognosis of only around 30 % [70, 71], we speculate that such an aggressive tumour might be able to affect many more CpG sites in the genome than other tumours, including those analysed with the here used age estimation tool.

Although this study provides a more comprehensive analysis of the influence of cancer on forensic age estimation tools than previous studies, there are some noteworthy limitations. It was difficult to obtain samples for the cancer cohort from individuals under the age of 40 years. Although our cancer cohort included individuals from the age of 25 years onwards, more samples from older individuals were available which skewed even the u75 cohort towards older ages compared to the control cohort (mean chronological age u75 cancer cohort = 59.37 years, mean chronological age control cohort = 46.12 years). In addition, this study aimed at investigating as many different cancer entities as possible. Consequently, the hematologic tumour subgroup was considerably smaller than the solid tumour subgroup. More samples in this group as well as in the solid tumour group might have led to more pronounced statistically significant differences in age estimates between the cancer groups and the controls.

In conclusion, this study has shown that forensic age estimation tools, when including the same markers and target sites as those investigated here, will likely reflect age acceleration caused by cancer. If an age estimation tool is unknowingly used on a stain from a cancer patient, the estimation accuracy might be slightly reduced compared to healthy individuals. However, our study only reports a small increase in estimation error in all entities except for highly aggressive cancers (represented by CLLs and AMLs in this study). Therefore, it might be an option to mention extreme medical conditions like aggressive hematologic tumours as potential limitations in a case report without requiring any further adaptations. This study is in line with previous studies that investigated environmental influences on the accuracy of forensic age estimation tools and have shown that the here targeted markers are relatively robust against such influences. Conclusively, this study contributes to the growing body of knowledge on factors influencing forensic age estimation and may provide guidance for casework applications.

## MATERIALS AND METHODS

### Study cohort and sample collection

The cancer cohort of this study consisted of venous blood samples from 100 participants with various cancerous diseases, collected during routine check-ups at the Onkozentrum Zurich, Switzerland (age range [y] = 25.46 - 93.15, mean = 65.88; n male = 56, n female = 44). For the control cohort, venous blood was collected from 102 healthy participants during blood donations at the Blood Donation Centre Zurich, Switzerland (age range [y] = 20.87 - 73.16, mean = 46.12; n male = 78, n female = 24). In order to be considered for blood donation, blood donors must not have a current malignancy and, in case of past occurrences of a malignancy, must have been declared cured for at least five years. Individuals with a hematologic tumour (past or present) are excluded from donating blood. All blood samples were stored at -20° C until DNA extraction.

### Ethical approval

This study was approved by the Ethics Committee of the University of Zurich (BASEC-No. 2023-00196) and all participants from the Onkozentrum Zurich provided an informed consent. Participants in the healthy control cohort had provided a general consent at the time of blood donation to the general use of their samples for research. All samples were anonymized. For the cancer cohort, information on the cancer entity, current treatment, previous treatment, chronological age and sex was collected. For the control cohort, only chronological age and sex were known.

### DNA extraction, DNA quantification and bisulfite conversion

300 μl of blood per individual were extracted with the Promega Maxwell® RSC48 instrument (Promega, Madison, WI, USA) according to the Maxwell RSC Whole Blood DNA protocol. DNA quantity was measured with the Quantus Fluorometer (Promega) and then bisulfite conversion was performed on 100 ng of extracted DNA with the MethylEdge® Bisulfite Conversion System (Promega).

### Library preparation and sequencing

Sequencing of all samples was performed as published by Woźniak et al. [7]. 8 μl of bisulfite converted DNA was used in the multiplex PCR reaction. Paired-end sequencing of 201 cycles was performed for all samples with a MiSeq FGx Reagent Micro or MiSeq FGx Reagent kit (Qiagen, Hilden, Germany) on a MiSeq FGx instrument (Illumina, San Diego, CA, USA). Sequence alignment from the fastq files was done with a custom Python script using the bwa aligner for bisulfite-treated sequences [72]. Bam files were created and indexed with Samtools [73, 74]. Read counts were extracted from the bam files based on unmerged reads. For the calculation of the age estimates, the dongle containing the VISAGE software, that is available on request from the VISAGE consortium, was used [11]. Run quality was assessed with custom R scripts checking for: 1) bisulfite conversion efficiency ≥ 90 %, 2) base misincorporation rate ≤ 2 %, 3) read depth of at least 1000 reads at each target site, and 4) a visual inspection of the alignment in the Integrative Genomics Viewer (IGV) [75].

### Data analysis

Data analysis was performed in R. Beta values at the target sites were calculated by dividing the reads corresponding to the methylated cytosines by the sum of the methylated and unmethylated reads. Age estimates were generated with the statistical model for blood [7]. PCA plots on the beta values of the target sites, normalized for chronological age with the *lm* function from the package *stats* [76], were generated with the R function *prcomp* [76] and heat maps were generated with the package *pheatmap* [77]. Statistical comparisons and graphical representations were performed with the R packages *stats*, *ggplot2* and *tidyverse* [76, 78, 79].

## Supplementary Material

Supplementary Figures

Supplementary Tables
