# Supplementary material for: The influence of cancer on a forensic age estimation tool
Source: Aging (Albany NY). 2025 Jul 17;17(7):1679–701. doi: 10.18632/aging.206281 (PMC12339023; doi:10.18632/aging.206281)
Supplement: Supplementary Figures [file aging-17-206281-s001.pdf]

SUPPLEMENTARY FIGURES

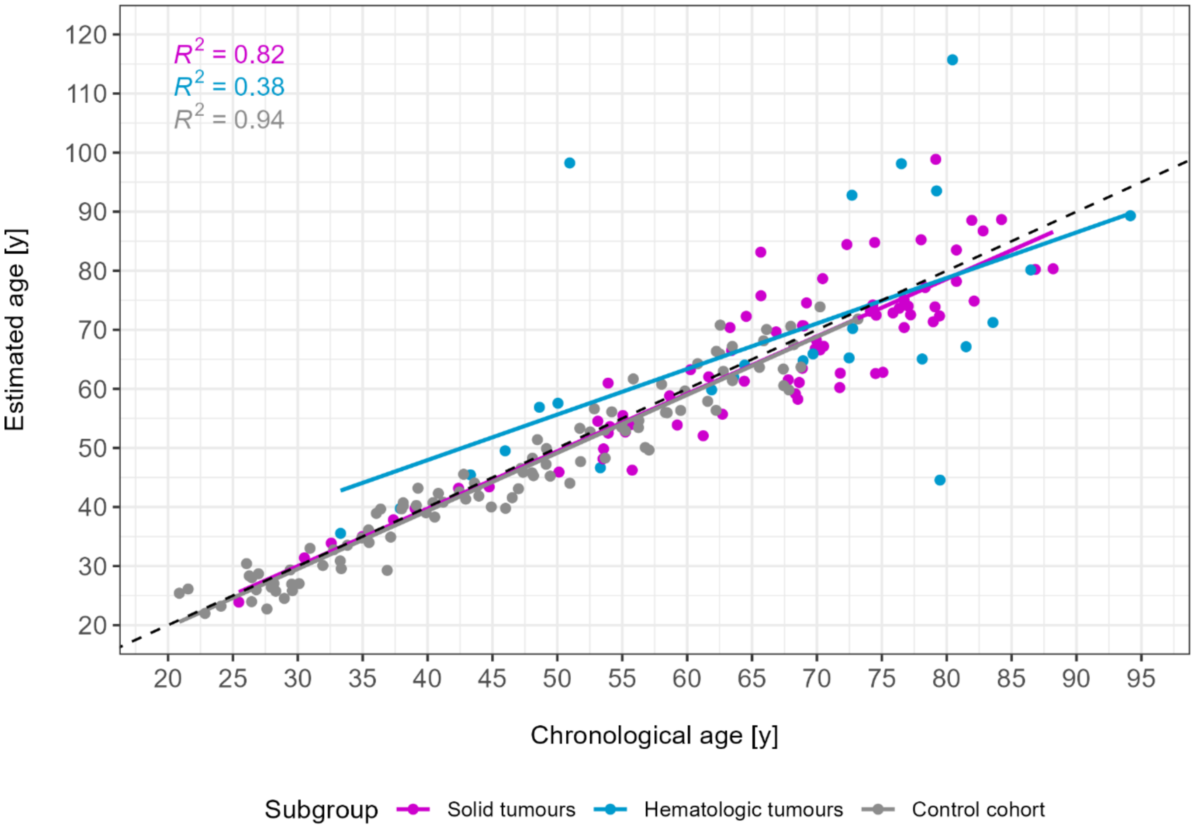

Supplementary Figure 1. Age estimates of the study cohort (n = 202). Solid tumours are depicted in magenta (n = 75), hematologic tumours in blue (n = 25) and the control cohort in grey (n = 102). R^2 of each subgroup are depicted in the upper left corner. The grey dotted line is the line of identity. MAE in years: 4.98 (solid tumours), 11.14 (hematologic tumours), 2.72 (control cohort).

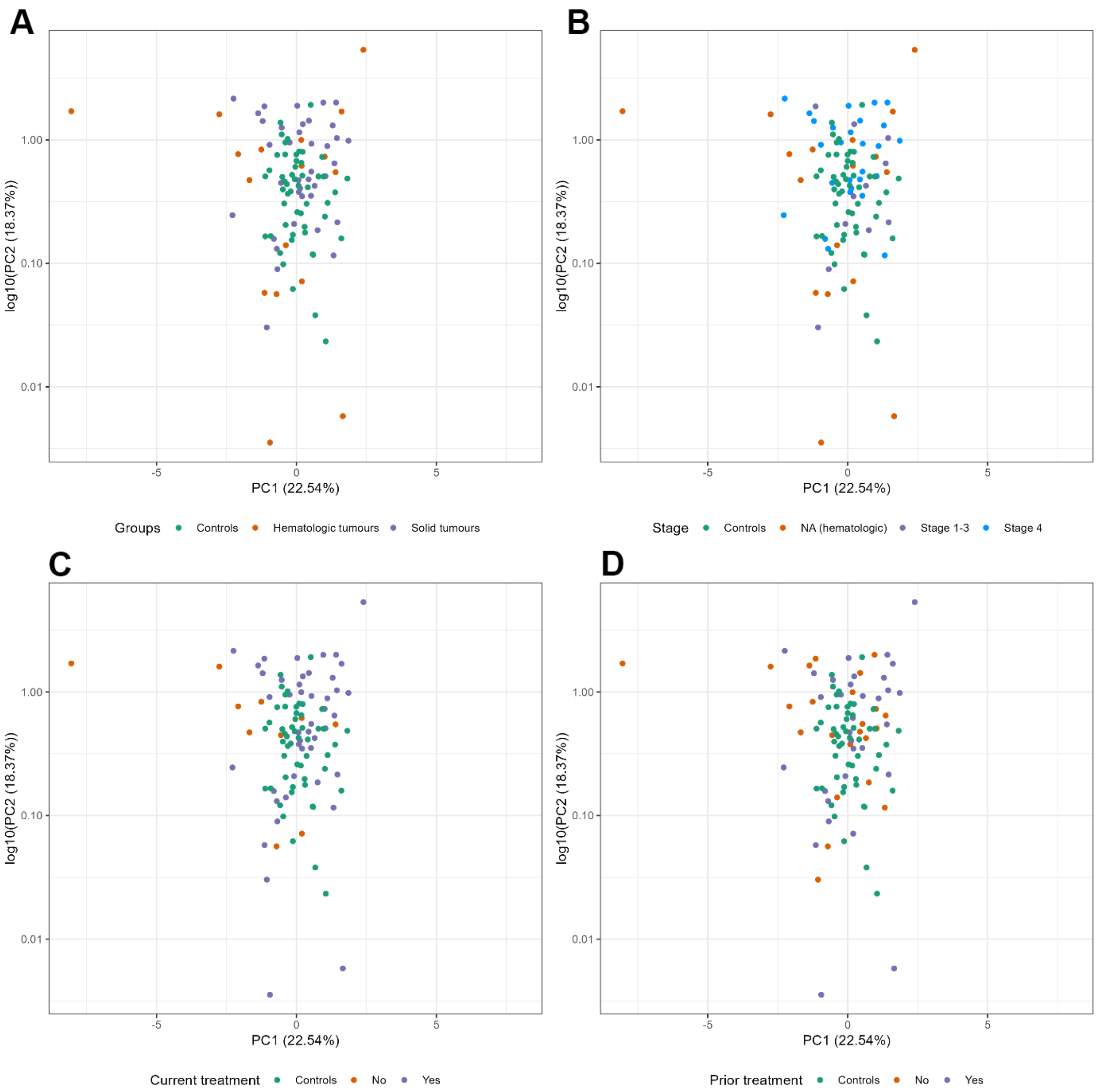

Supplementary Figure 2. PCA plots on the six target CpG sites of all samples. The beta values are normalized with linear regression to the respective chronological age of each individual. Each plot is colored according to a different meta data: grouping (**A**), stage (**B**), current treatment (**C**) and prior treatment (**D**). PC2 was log10 transformed for easier visual representation.

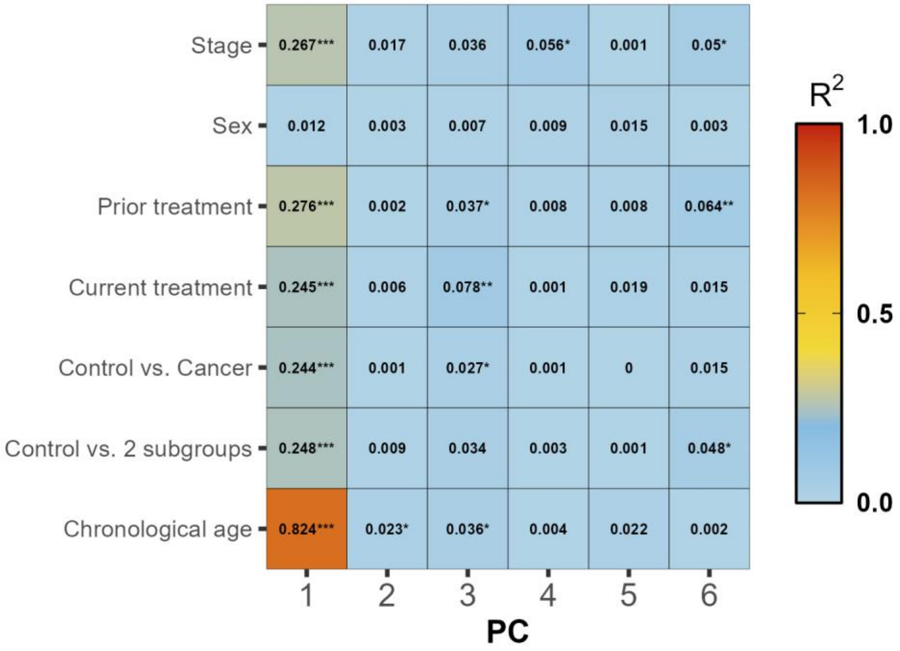

Supplementary Figure 3. Variable association of methylation beta values of all 44 sequenced CpG sites with available meta data. * p-value ≤ 0.05, ** p-value ≤ 0.01, *** p-value ≤ 0.001.

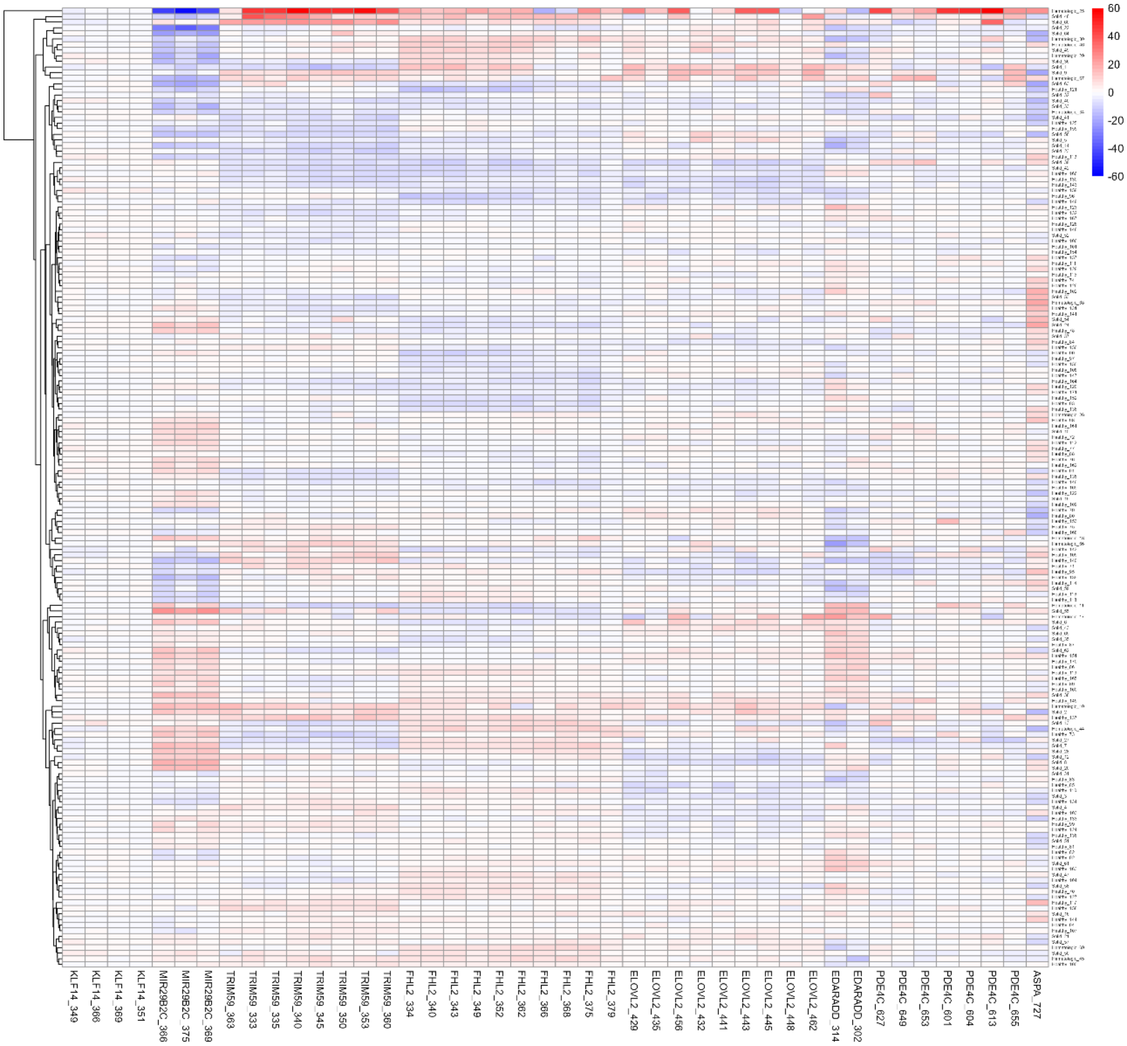

Supplementary Figure 4. Heatmap of methylation beta values of all 44 sequenced CpG sites. One sample clustered separately from all other samples (Cancer042).
