# Supplementary material for: The influence of cancer on a forensic age estimation tool
Source: Aging (Albany NY). 2025 Jul 17;17(7):1679–701. doi: 10.18632/aging.206281 (PMC12339023; doi:10.18632/aging.206281)
Supplement: Supplementary Tables [file aging-17-206281-s002.pdf]

SUPPLEMENTARY TABLES

Supplementary Table 1. Age and sex distribution of control and cancer cohorts.

	Control cohort			Cancer cohort		
**Age category [y]**	**Male (n)**	**Female (n)**	**Total (n)**	**Male (n)**	**Female (n)**	**Total (n)**
20.0 - 29.9	10	8	18	1	0	1
30.0 - 39.9	14	6	20	6	1	7
40.0 - 49.9	19	4	23	3	2	5
50.0 - 59.9	16	5	21	8	8	16
60.0 - 69.9	17	1	18	13	13	26
> 70	2	0	2	25	20	45

Supplementary Table 2. Statistical comparisons of the chronological age distributions of the three study cohorts.

Sample group comparison	p-value
Control vs. hematologic	7.753*10−7
Control vs. solid	3.659*10−14
Solid vs. hematologic	1.000

Dunn test with bonferroni correction.

Supplementary Table 3. Logistic regression analysis on the u75 study cohort and control samples.

Tested variables	p-value
(Intercept)	$3.53*10^{-8}$
Absolute age estimation error	0.06772
Chronological age	$5.93*10^{-9}$
Sex	0.00503

Dependent variable: disease status (healthy/cancer), independent variables: absolute estimation error, chronological age, sex.

Supplementary Table 4. Percentage of cancer samples above the MAE/RMSE of the control cohort per age category.

Age category	Cancer subgroup	% of samples above MAE	% of samples above RMSE
20.0 - 29.9	Solid	-	-
30.0 - 39.9	Solid	60	40
40.0 - 49.9	Solid	50	50
50.0 - 59.9	Solid	46	31
60.0 - 69.9	Solid	48	38
70.0 - 74.9	Solid	55	55
> 75.0	Solid	45	32
20.0 - 29.9	Hematologic	-	-
30.0 - 39.9	Hematologic	50	50
40.0 - 49.9	Hematologic	33	33
50.0 - 59.9	Hematologic	33	33
60.0 - 69.9	Hematologic	40	40
70.0 - 74.9	Hematologic	33	33
> 75.0	Hematologic	33	33

Supplementary Table 5. Mean errors (ME) per age category in all three subgroups.

Age category [y]	Control	Solid	Hematologic	p-value SvsC	p-value HvsC
20.0 - 29.9	-0.397	-1.56	-	0.842	-
30.0 - 39.9	-0.178	0.676	2.05	0.575	0.312
40.0 - 49.9	-1.35	-0.280	4.65	0.427	0.003*
50.0 - 59.9	-1.55	-1.94	16.0	0.780	0.271
60.0 - 69.9	-0.361	-0.111	-2.40	0.813	0.403
70.0 - 74.9	1.15	-1.09	3.44	0.641	0.800
> 75.0	-	-1.67	-1.63	-	-

* p-value < 0.05.

Control = control group, Solid = Solid tumours, Hematologic = Hematologic tumours. SvsC = Solid vs. Controls, HvsC = Hematologic vs. Controls.
